# Analysis of serum protein glycosylation by a differential lectin immunosorbant assay (dLISA)

**DOI:** 10.1186/1559-0275-10-12

**Published:** 2013-09-09

**Authors:** Danni Li, Hanching Chiu, Hui Zhang, Daniel W Chan

**Affiliations:** 1 Department of Pathology, Johns Hopkins University, Baltimore, MD, USA; 2 Department of Lab Medicine and Pathology, University of Minnesota, Twin Cities, 420 Delaware St SE, MMC 609, Mayo Building Room D250-1, Minneapolis, MN 55414, USA

**Keywords:** Lectin immunosorbant assay, LISA, Glycosylation change, TIMP-1, UEA, Fucosylation

## Abstract

**Background:**

Lectin immunosorbant assays (LISAs) have been widely used for analyzing protein glycosylation. However, the analysis of serum samples by LISAs could suffer from high sample-dependent background noise. The aim of this study is to develop a differential lectin immunosorbant assay (dLISA) with reduced background interferences.

**Methods:**

For the analysis of protein glycosylation, dLISA establishes a dose–response curve for every serum sample. The sample is split into five aliquots. Four aliquots undergo differential removal of the glycoprotein of interest by immunoprecipitation. Then, all five aliquots are subject to two measurements: protein by immunoassay and protein glycans by LISA. A dose–response curve is established by plotting glycans signals on the y-axis and protein levels on the x-axis for all the aliquots. Slope of the curve, calculated by linear progression analysis and expressed as fluorescence per concentration of protein, is used for the measurement of protein glycosylation in the serum sample.

**Results/conclusions:**

To demonstrate the feasibility of the dLISA approach, we used recombinant, fucosylated tissue inhibitor of metallopeptidase 1 (TIMP-1) as the target glycoprotein. Magnetic beads based TIMP1 immunoassay and TIMP-1 UEA LISA were developed for the measurement of TIMP1 protein and terminal α1, 2 fucosylated glycans on TIMP1, respectively. Serum samples supplemented with differentially fucosylated recombinant TIMP-1 were used to demonstrate that the slopes measured the TIMP-1 fucosylation, and were less prone to background interference.

## Introduction

Aberrant glycosylation of proteins has been implicated in many human diseases [1-3]. To aid in the diagnosis and prognosis, as well as in the understanding of these diseases at the molecular levels, there have been many research initiatives focusing on the development of analytical tools for effective analyses of subtle, yet biological significant, glycosylation changes [4-9]. One of the most informative and sensitive detection techniques for the analysis of glycans and glycoproteins is mass spectrometry (MS). Analytical tools combining MS with separation and enrichment techniques such as hydrophilic interaction chromatography (HILIC) and immunoaffinity enrichment are expected to provide a wealth of information on glycosylation changes that would allow a better understanding of the biological attributes of glycoproteins [10-13].

**Figure 1:**
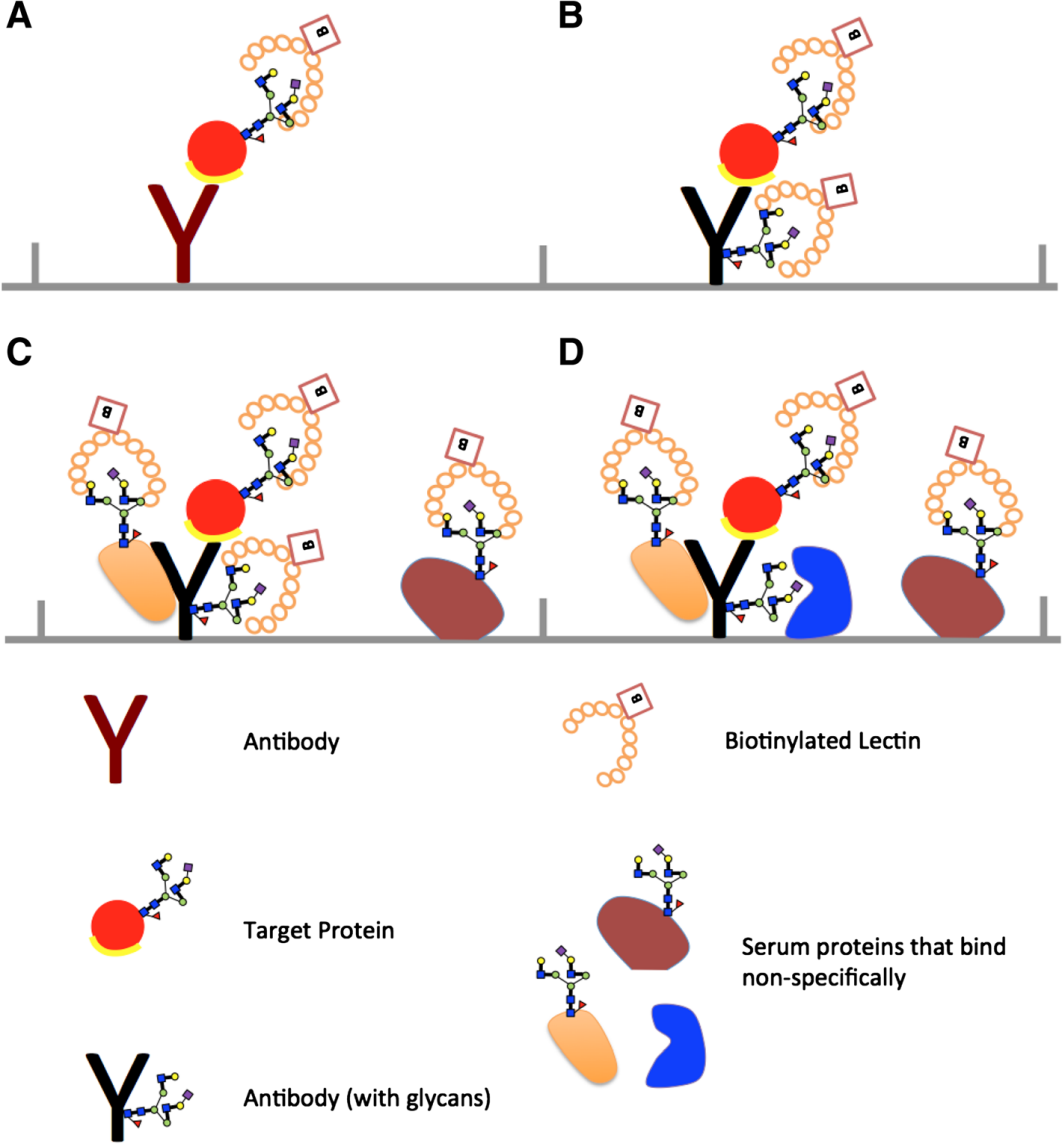
**Schematic representation of LISAs.** In LISAs, antibody captures the protein of interest and lectin detects the glycan structures on the protein (Figure 1**A**). Lectins would bind to antibodies (Figure 1**B**) and non-specific bound proteins (e.g., to the antibody and/or to solid surface that the antibody was adhered to) (Figure 1**C**-**D**).

Complementary to the MS based techniques are affinity-based techniques for the analysis of glycosylation changes, such as lectin immunosorbant assays (LISAs) [4,6,14-16]. LISAs are similar to enzyme-linked immunosorbant assays (ELISA) except that lectins are used as probes for detecting glycan structures (Figure 1A). The potential advantages of LISAs are several. First, LISAs are easy to set up. They can share the same instrument as the existing proteins immunoassays. Second, LISAs, when developed together with protein immunoassays, could generate integrated protein-glycan information [17]. Third, LISAs provide easy-to-interpret information that does not require sophisticated bioinformatics tools and supporting database. Lastly, due to lectins’ broad specificities, LISAs could provide additional information on all the glycans that the lectins bind to and resulting in better sensitivity for the detection of glycosylation changes of proteins.

LISAs have been used in the discovery and validation of glycosylation of proteins as biomarkers [4,6,14-16]. Despite the potential advantages, LISAs have not been widely exploited. In LISAs, lectins could bind to antibodies since antibodies are glycoproteins, resulting in high backgrounds that could reduce sensitivity (Figure 1B). Several ways of blocking the lectin-antibody binding have been used in LISAs, such as (i) enzymatic release of the glycans off the antibody and (ii) oxidation of the glycans followed by derivatization with di-peptides. Although these treatments were able to reduce the background signals, reduction varied by lectin [4,16,18]. For some lectins (e.g., Pisum savitum Lectin and Lens Culinaris Agglutinin), background signals after blocking were still too high [16]. Lectins could also bind to non-specific bound proteins (e.g., to the antibody and/or to solid surface that the antibody was adhered to) (Figure 1C-D). This could be problematic, especially when serum specimens are used, because the majority of serum proteins are glycosylated. The non-specific bound proteins could be reduced by the careful selection of blocking conditions. However, they could not be completely eliminated. Furthermore, the non-specific binding may vary from sample to sample. The sample-dependent background introduces significant variations in the signal generated by LISAs, making the comparison of glycosylation changes of proteins unreliable. In this study, we developed a dLISA approach for the analysis of protein glycosylation in serum that was less prone to background interference.

## Materials and methods

### Reagents

TIMP-1 capture antibody, TIMP-1 biotinylated detection antibodies, and recombinant TIMP-1 protein were purchased from the R&D Systems (Minneapolis, MN). Bio-Plex Pro™ magnetic COOH beads, Amine Coupling Kits, and Cytokine Assay Kits were purchased from Bio-Rad Laboratories (Hercules, CA). Biotinylated Ulex europaeus agglutinin (UEA) was purchased from Vector Labs (Burlingame, CA). Dynabeads Antibody Coupling Kit was purchased from Invitrogen (Carlsbad, CA).

### Clinical specimens

Serum samples from cancer and non-cancer patients (breast cancer, colon cancer, hepatocellular carcinoma, ovarian cancer, lung cancer, and prostate cancer) were obtained from the Serum Bank at the Center for Biomarker Discovery and Translation, Johns Hopkins University (Baltimore, MD) with the approval from the institutional review board.

### TIMP-1 immunoassay

TIMP-1 immunoassay was developed using the BioRad Cytokine Assay Kit (Hercules, CA). TIMP-1 capture antibody was coupled to Bio-Plex Pro™ magnetic COOH beads using the BioRad Amine Coupling Kit. The coupling was then validated using biotinylated goat anti-mouse IgG antibodies (Sigma-Aldrich, St. Louis, MO) to ensure binding of the capture antibody to the beads. After the validation, the beads were counted, and stored in the storage buffer at 4°C. For the TIMP-1 immunoassay, 2500 of the beads (per well for a 96-well plate) were incubated with 50 μL of a serum sample diluted in the Sample Diluent (provided in the BioRad Cytokine Assay Kit) at room temperature for 60 minutes. After the incubation, the beads were washed and incubated with 25 μL of 2 μg/mL biotinylated TIMP-1 detection antibody diluted in the Detection Antibody Diluent (provided in the Cyotkine Assay Kit) at room temperature for 30 minutes. Then the beads were washed again and incubated with 50 μL of 2 μg/mL streptavidin-phycoerytherin diluted in the Assay Buffer (provided in the Cyotkine Assay Kit) at room temperature for 10 minutes before analysis using the Bioplex 200 System (BioRad, Hercules, CA). Bioplex 200 uses 635 nm solid-state Laser to excite the fluorescent dyes inside the magnetic COOH beads to provide bead classification and assay identification information, and uses 532 nm Nd-Yag Laser to excite the phycoerytherin dye to generate a reporter signal. Recombinant TIMP-1 was used as standard. Concentrations of 0, 0.02, 0.1, 0.39, 1.56, 6.25, and 25 ng/mL of recombinant TIMP-1 were prepared in the Standard Diluent (provided in the Cytokine Assay Kit) as calibrators for establishment of a calibration curve for TIMP-1 protein quantification.

### TIMP-1 UEA LISA

TIMP1 UEA LISA was established the same way as the TIMP1 immunoassay except that 20 μg/mL of biotinylated UEA was used for detection.

### Immunoprecipitation of endogenous TIMP-1 in serum

Immunoprecipitation of endogenous TIMP-1 in serum was achieved using Dynabeads coupled with TIMP1 capture antibody. The coupling of the TIMP-1 capture antibody to the Dynabeads was performed using the Dynabeads Antibody Coupling Kit. For the immunoprecipitation, every 10 μL of serum was mixed with 5 μL of the coupled Dynabeads. The amount of beads (5 μL) was empirically determined to be the sufficient for the complete removal of endogenous TIMP-1 from serum samples. The mixture was then incubated on a rotator overnight at 4°C. For partial removal of the endogenous TIMP-1, fewer beads (e.g., 3uL) would be used.

### Production of the differential fucosylated recombinant TIMP-1

Four microliter of the recombinant TIMP-1 (10 μg/mL prepared in PBS + 1% BSA buffer) was mixed with 4 μL of α1, 2 fucosidase (Catalog# P0724, New England Biolabs, Ipswich, MA). The mixture (8 μL) was then diluted 5 times in the 32 μL of the Reaction buffer (50 mM Sodium Citrate, 100 mM Sodium Chloride, pH 6.0) for incubation of 0, 15, 30, or 45 minutes with shaking at 37°C. After the incubation, each mixture was spiked into a serum aliquot. Presence of large quantities of glycoproteins in serum would stop the enzymatic digestion of the TIMP-1 by α1, 2 fucosidase. The serum aliquot did not contain endogenous TIMP-1, which was immuno-depleted. The immuno-depletion was confirmed using the TIMP-1 immunoassay.

### Data analysis

Calibration curve for the TIMP-1 immunoassay was established using the 5-parameter nonlinear regression model in Bio-Plex Manager™ 6.0. Protein concentrations were calculated using the calibration curve and reported by Bio-Plex Manager™ 6.0. For linear regression and statistic analysis of the dose–response curves, we used Graphpad Prism 5.04. Limit of detection of the TIMP-1 immunoassay, determined using 3 times of standard deviation of the zero calibrator over six measurements, was 0.01 ng/mL. Reproducibility of the TIMP-1 immunoassays, determined using the coefficient variance of the calibrators at concentrations of 0.02, 0.1, 0.39, 1.56, 6.25, and 25 ng/mL, was less than 20%.

## Results

### Overview of the dLISA approach

**Figure 2:**
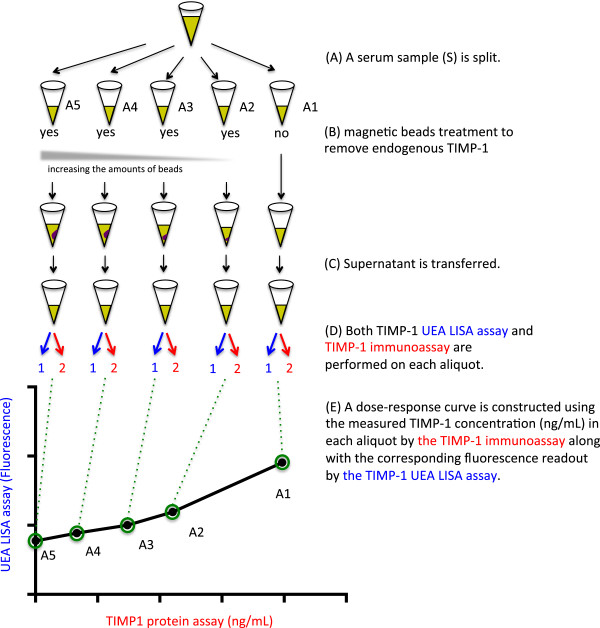
**Schematic representation of the dLISA approach. (A)** A serum sample is split into five aliquots (A1 to A5). **(B)** Four of these aliquots are treated with increasing amounts of magnetic beads coupled with TIMP-1 antibody to gradually reduce the amount of endogenous TIMP-1 present. For aliquot A5, there should be sufficient beads used for complete removal of the endogenous TIMP-1. **(C)** After the magnetic beads treatment, the beads were separated using a magnetic separator and the supernatant from each aliquot is transferred. **(D)** Both TIMP-1 UEA LISA assay and TIMP-1 immunoassay are performed on each aliquot. **(E)** A dose–response curve is constructed using the measured TIMP-1 concentration (ng/mL) in each aliquot by the TIMP-1 immunoassay along with the corresponding fluorescence readout by the TIMP-1 UEA LISA assay.

Using TIMP-1 fucosylation as an example, the dLISA approach of measuring glycosylation of serum proteins is described in Figure 2. A serum sample (S) is splitted into five aliquots (A1 to A5) with equal volume. Magnetic beads coupled with TIMP-1 antibodies are used for the removal of endogenous TIMP-1 in these aliquots: A1 is left untreated, whereas A2 to A5 are treated with increasing amounts of magnetic beads for the differential removal; the amount of beads used for A5 is sufficient for the complete removal of the endogenous TIMP-1. After the treatments, all the five aliquots are subject to the measurement by two methods: TIMP-1 immunoassay and TIMP-1 UEA LISA assay. The immunoassay is used to determine TIMP-1 protein levels in these aliquots, which should have decreasing levels of TIMP1 from A1 to A5; the TIMP-1 UEA LISA assay is used for the detection of UEA-associated, α1, 2 fucosylated glycans. As explained previously, LISA assays are prone to interferences. To tease out the portions of the signals that are specific for TIMP-1, the TIMP-1 UEA LISA signals of A1 to A5 are plotted on the y-axis against their respective TIMP-1 protein levels on the x-axis to establish a dose–response curve. Linear progression analysis determines the slope of the dose–response curve, which is expressed as fluorescence (A.U.) per TIMP-1 concentration (ng/mL). The slope reflects the changes in the TIMP-1 UEA LISA signal as a result of changes in TIMP-1 concentration (ng/mL).

Although LISA assay appears to be similar to dLISA assay, they are different in several ways: (1) LISA assay is performed as a single measurement, whereas dLISA assay establishes a dose–response curve based on measurement of five aliquots; (2) LISA assay measures both protein abundance as well as protein glycosylation, both of which are combined together and reported as a single read out; on the other hand, the dLISA approach includes protein immunoassay, separating protein abundance from protein glycosylation, and therefore, specifically measures protein glycosylation; (3) Because it differential removed the target protein using immunoprecipitation, the dLISA approach is more specific for the target protein; (4) The dLISA approach also includes measurement of the background signal through the aliquot A5. This helps to identify serum samples with potential high background signal that interferes with LISA assay, and thus it demonstrates the advantages of the dLISA approach with reduced interferences.

### Correlation of the slope with serum TIMP-1 fucosylation

**Figure 3:**
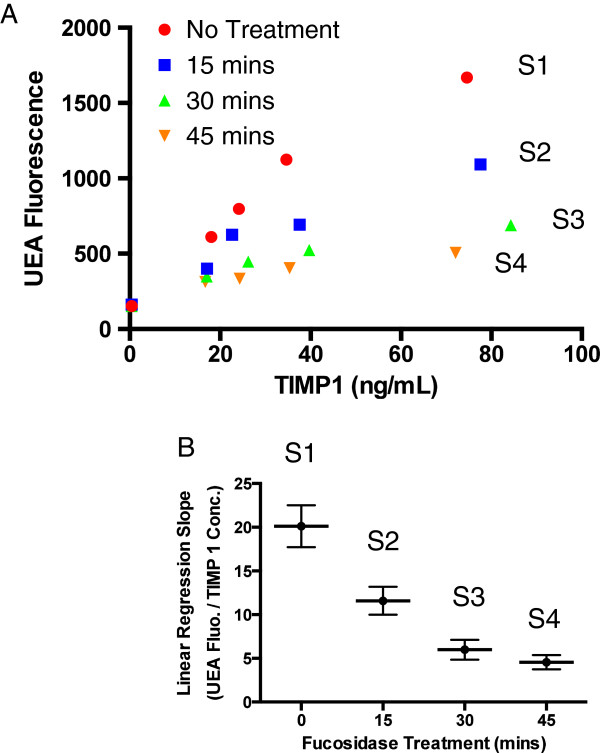
**Slopes of the dose response curves established using the dLISA approach correlated with TIMP-1 fucosylation. (A)** Dose–response curves of serum samples spiked with differentially fucosylated recombinant TIMP-1. Recombinant TIMP-1 was treated by α1, 2 fucosidase at different length of time (0, 15, 30, and 45 minutes) before they were spiked into with four serum aliquots. These aliquots came from the same pool of sera with endogenous TIMP-1 immuno-depleted (confirmed by the TIMP-1 immunoassay). After the recombinant protein spiked in, these serum samples underwent the dLISA approach. **(B)** A graph presentation of linear regression slopes of the dose–response curves versus the length of fucosidase treatment (minutes).

We demonstrated that the slopes of the dose response curves correlated TIMP-1 fucosylation using recombinant TIMP-1. The NS0 cell line derived recombinant TIMP-1 (R&D Systems, Minneapolis, MN) contains terminal α1, 2 fucosylated glycans associated with UEA [19]. First, we prepared 4 serum samples with differentially fucosylated TIMP-1 by treating the recombinant TIMP-1 with α1, 2-fucosidase at various time lengths (0, 15, 30, and 45 mins). α1, 2-fucosidase digests off α1, 2 fucose, to which UEA has specificity towards. The longer the treatment was, the more α1, 2 fucose was cut off from TIMP-1, and the less fucosylated the TIMP-1 became. Therefore, the four treatment conditions (0, 15, 30, and 45 mins) created recombinant TIMP-1 proteins with 4 differential levels of fucosylation. These proteins were then spiked into four serum aliquots (S1 to S4) that did not contain endogenous TIMP-1 (Endogenous TIMP-1 in these aliquots was immuno-depleted and confirmed using the TIMP-1 immunoassay). Second, each of these four samples (S1 to S4) underwent the dLISA approach. Curves and slopes of these four samples are illustrated in Figures 3A and 3B, respectively. The level of fucosylation decreased in these samples from S1 to S4, so were the linear regression slopes, decreasing from 20 ±2, 12± 2, 6 ± 1, to 5± 1 (fluorescence/ (ng/mL of TIMP-1 conc.), respectively. Pearson correlation (r) between the lengths of the fucosidase treatments (mins) and the slopes was -0.96 (95% confidence interval of -0.99 to -0.05, one-tailed p value of 0.02), indicating that the slopes correlated with the levels of TIMP-1 fucosylation.

### Application of the dLISA approach to serum samples

**Figure 4:**
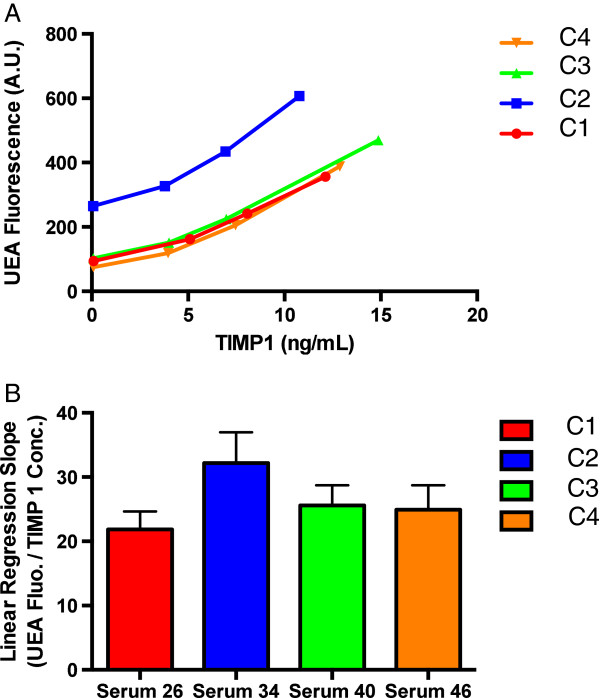
**Dose-response curves and slopes of the dLISA approach when applied to serum specimens with the same fucosylated TIMP-1 in different serum matrices. (A)** Dose–response curves of four samples with the same fucosylated TIMP1 in different matrices. For preparation of these specimens, we identified four individual serum samples. Each one of them was then treated for complete removal of endogenous TIMP-1 (confirmed by the TIMP-1 immunoassay). Recombinant TIMP-1 was added into each of the serum matrices. Then these serum samples underwent the dLISA approach. **(B)** A bar graph with linear regression slopes of the dose–response curves.

We applied the dLISA approach to serum samples in oder to demonstrate its advantages over LISA using 4 specimens (C1 to C4) with the same fucosylated TIMP-1 in different serum matrices. Figure 4A and 4B shows the dose–response curves and slopes of the dLISA approach, respectively. Signals of the LISA assay (or the TIMP-1 UEA LISA) for these specimens were shown by the y-axis of Figure 4A. The dLISA approach indicated that the slope of C2 was not statistically significant from that of C1, C3, or C4 (32 ± 5 for C2 vs. 22 ± 3 for C1, or 26 ± 3 for C3, or 25 ± 4 for C4), consistent with the fact that these specimens contain the same recombinant TIMP-1. However, the y-axis (Figure 4A) showed the higher LISA signals of C2 than those of C1, C3, or C4 at any given TIMP-1 protein concentrations (x-axis in Figure 4A), indicating that C2 contained TIMP-1 with higher level of fucosylation than that of C1, C3, or C4, inconsistent with the fact that these specimens contain the same recombinant TIMP-1. This was due to the higher background signal of C2 (265) than those of C1, C3, or C4 (94, 103, or 75, respectively), as indicated when TIMP-1 was completely removed in these specimens (when TIMP-1 protein concentration was 0 ng/mL). Therefore, we demonstrated that the dLISA approach was less prone to the background interference than LISA.

**Figure 5:**
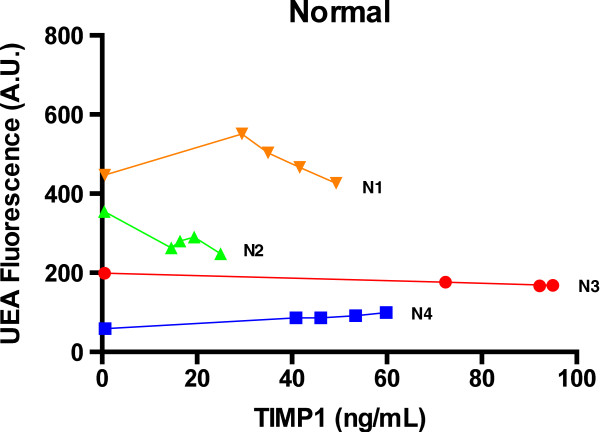
**Dose–response curves of serum samples from 4 healthy subjects.** Healthy serum samples 1, 2, 3, and 4, represented by the orange, green, red, and blue-colored lines, respectively. Each serum sample underwent the dLISA approach.

We also applied the dLISA approach to serum samples from normal and cancer conditions. Figure 5 showed the dose–response curves of the four normal serum samples N1, N2, N3, and N4. Serum N3 and N4 had the slopes of -0.3 ± 0.1, and 0.65 ± 0.04, respectively. Both slopes were close to zero, indicating that TIMP-1 in both sera N3 and N4 had little UEA fucosylation. The dose response curves of sera N1 and N2 showed that reduction in TIMP-1 protein increased the fluorescence of UEA detection in both serum N1 and N2. This indicated the existence of interferences with the LISA UEA assay in serum N1 and N2. Nevertheless, the slopes of N1 and N2, -0.1 ± 1.5, and -4 ± 1, respectively, were consistent with N3 and N4, that serum TIMP-1 had little UEA fucosylation. UEA fucosylation pattern of TIMP-1 were also analyzed in six different cancers (breast cancer, colon cancer, hepatocellular carcinoma, ovarian cancer, lung cancer, and prostate cancer). Linear regression slopes of their dose–response curves (shown in Table 1 and Additional file 1: Figure S1) were close to zero for breast cancer, colon cancer, hepatocellular carcinoma, and lung cancer, indicating that serum TIMP-1 had little or no UEA fucosylation in these cancer conditions.

**Table 1:** Linear-regression slopes of the dose–response curves of four individual serum samples of normal, breast cancer, colon cancer, HCC, ovarian cancer, lung cancer, and prostate cancer

**Slopes**							
**Specimen #**	**Normal**	**Breast cancer**	**Colon cancer**	**HCC**	**Ovarian cancer**	**Lung cancer**	**Prostate cancer**
1	-0.1 ± 1.5	-0.9 ± 0.1	0.09 ± 0.13	-1 ± 0	2 ± 0	-0.4 ± 0.3	2 ± 1
2	-4 ± 1	-2 ± 0	0.07 ± 0.22	-1 ± 0	0.5 ± 0.5	-0.6 ± 0.5	1 ± 0
3	0.7 ± 0.0	-1 ± 0	-0.5 ± 0.2	-0.3 ± 0.0	2 ± 0	2 ± 0	0.51 ± 0.04
4	-0.3 ± 0.0	-0.8 ± 0.3	0.8 ± 0.2	0.5 ± 0.1	2 ± 1	-0.2 ± 0.2	0.1 ± 0.2

## Discussion

TIMP-1 belongs to a family of tissue inhibitors of matrix metalloproteinases (MMPs) whose inhibitory activities play important roles in cellular homeostasis, tissue remodeling, and oncogenesis [20]. Expression of TIMP-1 was increased in cancer tissue in prostate [21]. TIMP-1 was reported to have prognostic and predictive value in breast cancer [22,23], and serum TIMP-1 was a predictor of survival outcomes in colorectal cancer [24]. In addition, TIMP-1 has been implicated in MMP-independent actions, such as synaptic plasticity of the central nervous system [25].

Aberrant glycosylation of TIMP-1 was implicated in cancer progression. Increased β1, 6 branching of N-glycans of TIMP-1, induced by GnT-V N-acetylglucosaminyltransferase, was closely correlated with invasive/metastatic potential of colon cancer cell WiDr [26]. Detection of TIMP-1 UEA α1, 2 fucosylation in prostate tissues was found to be superior to TIMP-1 protein in distinguishing aggressive and non-aggressive prostate cancer [17]. Whether detection of TIMP-1 UEA fucosylation in serum could help identify aggressive prostate cancer remained to be determined. We measured TIMP-1 UEA fucosylation in sera of prostate cancer and found little UEA fucosyaltion. The seemingly contradictory findings between prostate tissues and sera could be due to the source differences of TIMP-1. Presence of TIMP-1 in serum may be a combination of physiology (e.g., for carrying out their functions in circulation), tissues leakages (e.g., as a result of cell death or damage), and/or aberrant secretions (e.g., released from tumors and other disease tissues, presumably not as a result of a functional requirement) [27]. Given the potential sources of TIMP-1 in serum, if TIMP-1 from other sources were not fucosylated, the contribution of prostate tissue TIMP-1 would be too small to be detected. The same pattern was also observed in non-cancer sera and sera of other cancers such as breast, colon, lung, and ovarian, indicating that (1) TIMP-1 from other tissues may not be fucosylated, and (2) there was no difference in serum TIMP-1 fucosylation between non-cancer and cancers. This latter finding was consistent with Thaysen-Anderson et al., which showed no significant difference of TIMP-1 glycoprofiles between normal and colon cancer [9].

Ahn et al. showed that Leukocyte phytohemagglutinin (L-PHA) captured glycoforms of TIMP-1 in a pooled colon cancer serum was 5 times higher in abundance than that in a pooled non-cancer serum by mass spectrometric analysis [28]. We did not try TIMP-1 L-PHA LISA in serum because the recombinant TIMP-1 did not have L-PHA bound glycans [19] and therefore could not be used as the standard protein for the feasibility study. This points out a bigger issue in the field of glycobiology. In the future, development of protein engineering technology that allows additions of glycans of interest to proteins and produces highly purified protein glycoforms [29] would help solve this problem.

## Conclusions

Using recombinant TIMP-1 as the model, we determined a dLISA approach for the analysis of serum protein glycosylation that was less prone to potential interference of serum matrices. Applying the approach of analysis TIMP-1 fucosylation in serum samples, we found that serum TIMP-1 had little or no α1, 2 fucosylation in normal and many cancer conditions.

## Competing interests

The authors declare that they have no competing interests.

## Authors’ contributions

DL contributed to design of the experiments and drafted the manuscript. HC performed the experiments. HZ contributed to discussion of the experimental designs. DWC contributed to editing of the manuscript. All authors read and approved the final manuscript.

## Supplementary Material

Additional file 1: Figure S1 Dose-response curves of serum samples of breast cancer **(A)**, colon cancer **(B)**, HCC (C), ovarian cancer **(D)**, lung cancer **(E)**, and prostate cancer **(F)**. For each condition, there are four samples 1, 2, 3, and 4, represented by the orange, green, red, and blue-colored lines, respectively. Click here for file
