# Supplementary material for: Analysis of serum protein glycosylation by a differential lectin immunosorbant assay (dLISA)
Source: Clin Proteomics. 2013 Sep 9;10(1):12. doi: 10.1186/1559-0275-10-12 (PMC3847486; doi:10.1186/1559-0275-10-12)
Supplement: Additional file 1: Figure S1 — Dose-response curves of serum samples of breast cancer (A), colon cancer (B), HCC (C), ovarian cancer (D), lung cancer (E), and prostate cancer (F). For each condition, there are four samples 1, 2, 3, and 4, represented by the orange, green, red, and blue-colored lines, respectively. [file 1559-0275-10-12-S1.pptx]

## Slide 1
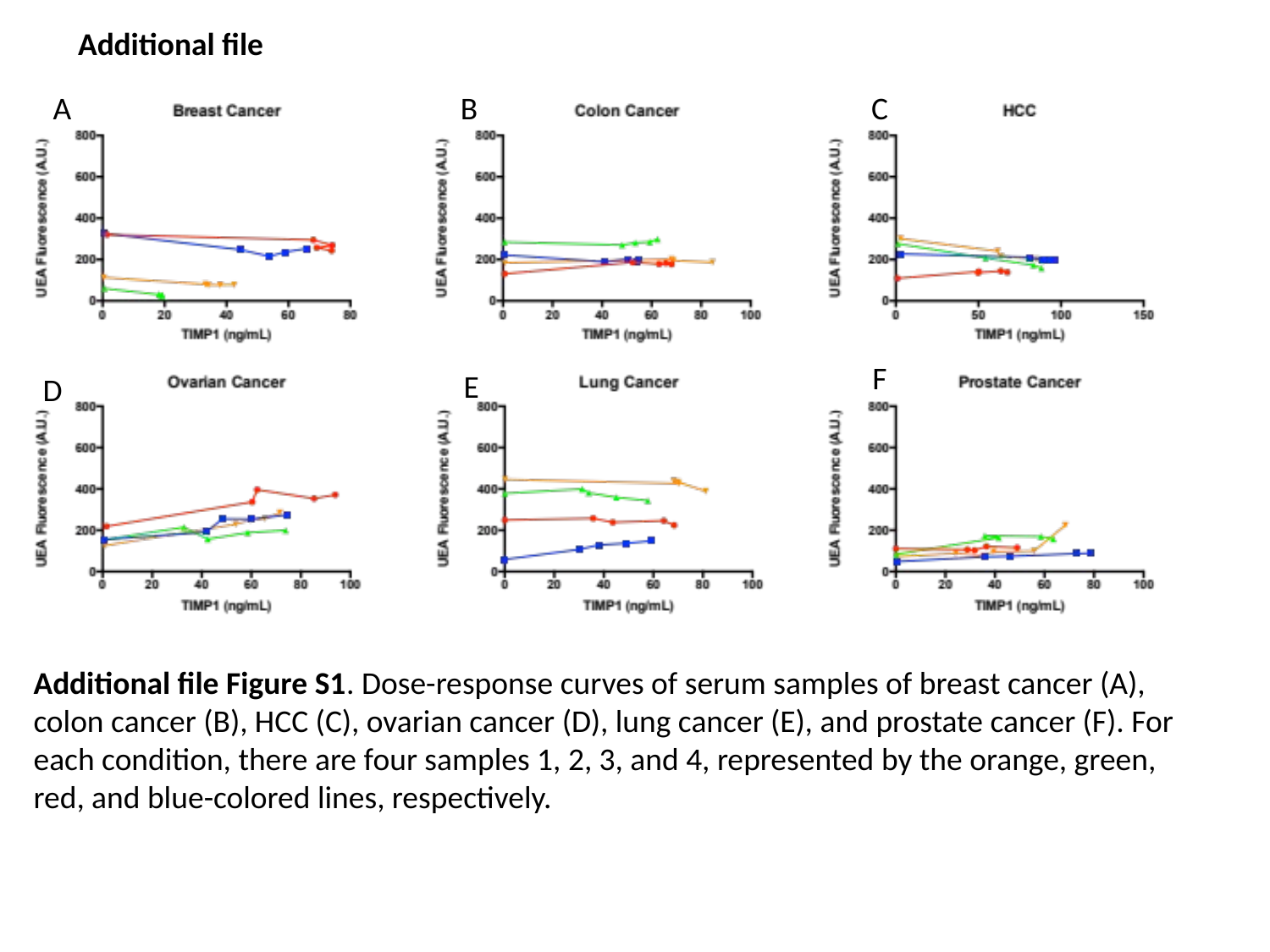

Additional file
A
B
C
F
E
D
Additional file Figure S1. Dose-response curves of serum samples of breast cancer (A), colon cancer (B), HCC (C), ovarian cancer (D), lung cancer (E), and prostate cancer (F). For each condition, there are four samples 1, 2, 3, and 4, represented by the orange, green, red, and blue-colored lines, respectively.
